# Group B Streptococcus CAMP Factor Does Not Contribute to Interactions with the Vaginal Epithelium and Is Dispensable for Vaginal Colonization in Mice

**DOI:** 10.1128/Spectrum.01058-21

**Published:** 2021-12-15

**Authors:** Mallory B. Ballard, Vicki Mercado-Evans, Madelynn G. Marunde, Hephzibah Nwanosike, Jacob Zulk, Kathryn A. Patras

**Affiliations:** a Department of Molecular Virology and Microbiology, Baylor College of Medicine, Houston, Texas, USA; b Medical Scientist Training Program, Baylor College of Medicine, Houston, Texas, USA; c Alkek Center for Metagenomics and Microbiome Research, Baylor College of Medicine, Houston, Texas, USA; University of Florida

**Keywords:** group B *Streptococcus*, *Staphylococcus aureus*, CAMP factor, vaginal colonization, biofilm

## Abstract

The Gram-positive pathogen group B Streptococcus (GBS) is a leading cause of neonatal bacterial infections, preterm birth, and stillbirth. Although maternal GBS vaginal colonization is a risk factor for GBS-associated adverse birth outcomes, mechanisms promoting GBS vaginal persistence are not fully defined. GBS possesses a broadly conserved small molecule, CAMP factor, that is co-hemolytic in the presence of Staphylococcus aureus sphingomyelinase C. While this co-hemolytic reaction is commonly used by clinical laboratories to identify GBS, the contribution of CAMP factor to GBS vaginal persistence is unknown. Using *in vitro* biofilm, adherence and invasion assays with immortalized human vaginal epithelial VK2 cells, and a mouse model of GBS vaginal colonization, we tested the contribution of CAMP factor using GBS strain COH1 and its isogenic CAMP-deficient mutant (Δ*cfb*). We found no evidence for CAMP factor involvement in GBS biofilm formation, or adherence, invasion, or cytotoxicity toward VK2 cells in the presence or absence of S. aureus. Additionally, there was no difference in vaginal burdens or persistence between COH1 and Δ*cfb* strains in a murine colonization model. In summary, our results using *in vitro* human cell lines and murine models do not support a critical role for CAMP factor in promoting GBS vaginal colonization.

**IMPORTANCE** Group B Streptococcus (GBS) remains a pervasive pathogen for pregnant women and their newborns. Maternal screening and intrapartum antibiotic prophylaxis to GBS-positive mothers have reduced, but not eliminated GBS neonatal disease, and have not impacted GBS-associated preterm birth or stillbirth. Additionally, this antibiotic exposure is associated with adverse effects on the maternal and neonatal microbiota. Identifying key GBS factors important for maternal vaginal colonization will foster development of more targeted, alternative therapies to antibiotic treatment. Here, we investigate the contribution of a broadly conserved GBS determinant, CAMP factor, to GBS vaginal colonization and find that CAMP factor is unlikely to be a biological target to control maternal GBS colonization.

## INTRODUCTION

The Gram-positive bacterium group B Streptococcus (GBS or Streptococcus agalactiae) is the leading infectious agent of early-onset neonatal sepsis (1) and is increasingly recognized as a cause of stillbirth (2) and preterm birth (3). GBS asymptomatically colonizes the vagina of ∼18% of pregnant women (4), and thus 21 million infants are exposed to maternal GBS at, or before, time of delivery (2). Approximately half of infants born to GBS-positive women become colonized (5), however, a subset of infants develop invasive GBS infections and subsequently 90,000 infants die annually (2). Because maternal GBS vaginal carriage is a critical risk factor for neonatal disease, many countries have implemented maternal screening and intrapartum antibiotic prophylaxis (IAP) for GBS-positive or at-risk mothers. These measures have reduced, but not eliminated, GBS early-onset disease, yet this antibiotic exposure comes with unintended consequences on the infant microbiota and may play a role in the propagation of antimicrobial resistance genes (6–9). Delineating the GBS components promoting successful mucosal colonization and dissemination of the maternal reproductive tract will provide insight into alternative non-antibiotic therapeutic development and identification of pregnancies at risk for fetal or neonatal mortality.

GBS possesses a number of unique features including beta-hemolytic activity (10), production of Granadaene, a red ornithine rhamno-polyene pigment (11), and production of a co-hemolysin, CAMP factor, named by its discovers Christie, Atkins, and Munch-Petersen (12, 13). Although beta-hemolytic activity and pigment production are associated with GBS vaginal persistence and uterine dissemination (14–16), the role of GBS CAMP factor in vaginal colonization is undefined. Since its discovery, GBS CAMP factor has been widely used as a clinical diagnostic microbiology test to identify GBS (17). CAMP factor, encoded by *cfb* (18), is an ∼25 kDa small molecule which oligomerizes to form pores in erythrocytes and other cell membranes rendered susceptible by various sphingomyelinases (19, 20) such as Staphylococcus aureus sphingomyelinase C (β-toxin). CAMP factor utilizes glycan moieties of GPI-anchored proteins as cell receptors for its C-terminal domain to drive oligomerization and pore formation through membrane insertion by the N-terminal domain (21, 22). Although originally described in GBS, *cfb* homologues have been identified in Streptococcus pyogenes at 67% identity (23), as well as and other Streptococci (23–25), Propionibacterium acnes (26), *Finegoldia* spp. (27), and Aspergillus *micronesiensis* (28). The frequent distribution of *cfb* homologues among pathogenic streptococci suggest that these factors may make substantial contributions to fitness for host colonization or pathogenicity.

Given the highly conserved nature of CAMP factor across GBS human and animal isolates (29), it has been hypothesized to be an important virulence factor. Immunoglobulin binding activity of GBS CAMP factor was reported (30), but subsequent studies failed to observe CAMP factor binding to IgG Fc fragments (31). Thus, its cytolytic properties remain its only known function. In other species, additional roles have been suggested, such as induction of vacuolation of macrophages, and reduced cell growth and phagocytosis in response to S. pyogenes CAMP factor (32). The contribution of CAMP factor to GBS-host interactions remains elusive. CAMP factor was found to be dispensable for GBS virulence in systemic infection (33), and purified CAMP factor was not toxic when administered intravenously in mice (30) unless a dose of ∼67.5 kU/kg was administered (34). It is possible that the lack of a CAMP factor effect in these models is due to the absence of host and/or bacterial sphingomyelinases to aid its co-hemolytic activity. When GBS is identified in polymicrobial sepsis, the most common coinfecting organism is S. aureus (35–39). Additionally, GBS and S. aureus are frequently coisolated from human vaginal samples (40–43) and from the nasopharynx of infants (44). We hypothesized that GBS CAMP factor may play an important role in GBS interactions with the host epithelium or endogenous microbiota, such as S. aureus, at the vaginal mucosa. In this study, we incorporated *in vitro* assays together with a murine model of GBS vaginal colonization to establish the contribution of CAMP factor using wildtype GBS and its isogenic CAMP-deficient mutant (Δ*cfb*) (33).

## RESULTS

### CAMP factor deficiency does not alter GBS growth, morphology, biofilm formation, or competition with S. aureus.

**FIG 1:**
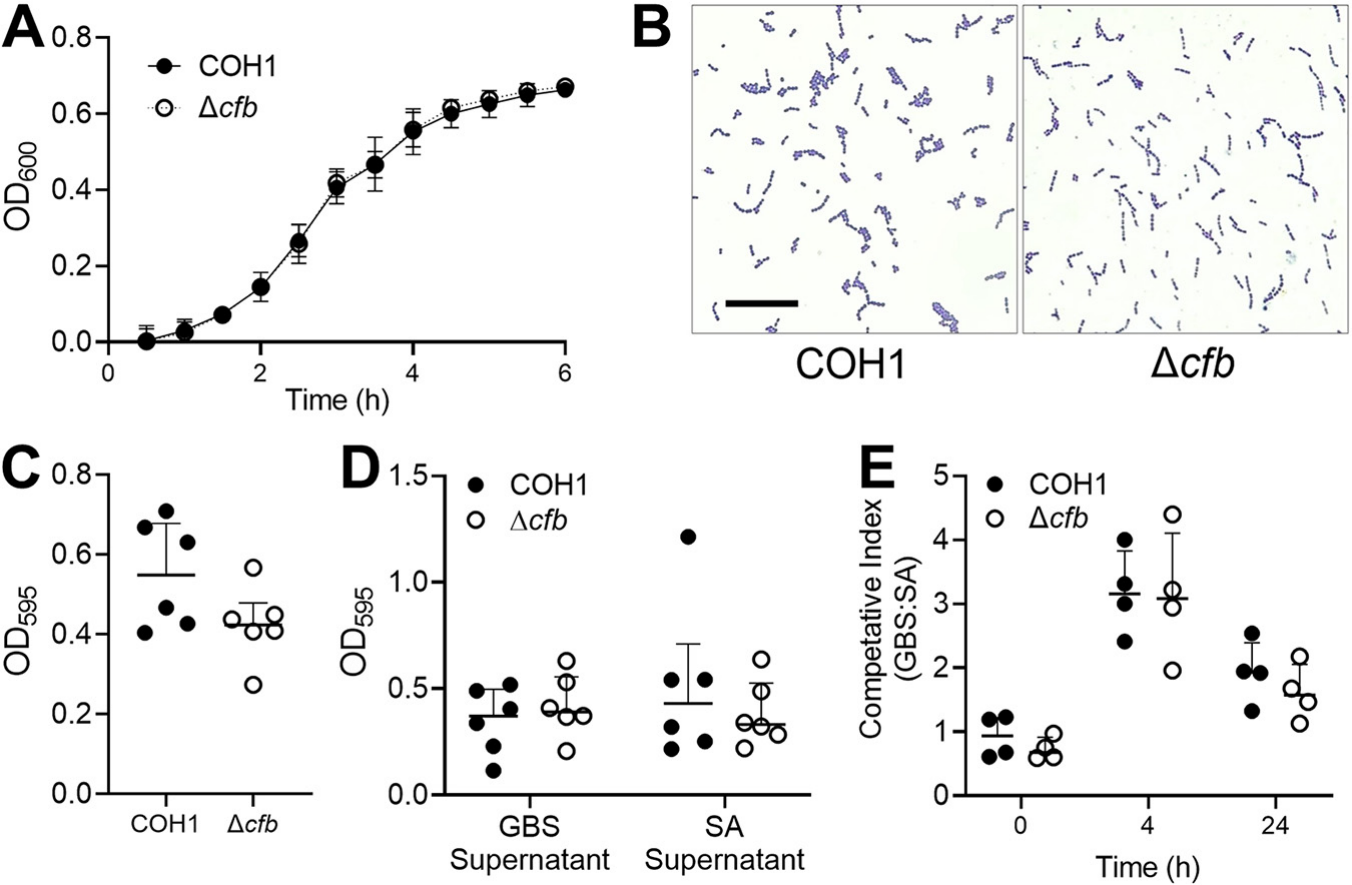
GBS CAMP factor is dispensable for GBS growth, morphology, biofilm production, and interspecies competition with S. aureus. (A) WT GBS COH1 and Δ*cfb* growth in THB over 6 h as represented by OD_600_ measurements every 30 min. (B) Morphology of log phase COH1 and Δ*cfb* cultures was assessed by Gram staining. Magnification  =  630×, scale bar  = 20 μm. (C) Biofilm production after 24 h was measured by crystal violet staining. (D) Biofilm production after 24 h in spent supernatant from either GBS cultures (self-supernatant from COH1 or Δ*cfb*) or S. aureus (SA) as measured by crystal violet straining. (E) COH1 and Δ*cfb* were grown singly or in combination with S. aureus (SA) in THB and viable CFU quantified a 0 h, 4 h, and 24 h. Shown is the competitive index which was calculated as the ratio of recovered GBS CFU/mL to S. aureus CFU/mL. All experiments were performed in technical duplicates with three to six independent experimental replicates. Individual points represent independent replicate mean with lines showing 95% CI (A) or independent replicates and lines showing median with interquartile range. Data were analyzed by two-way repeated measures ANOVA with Sidak’s multiple-comparison test (A, D, E), or Wilcoxon matched-pairs signed rank test (C). All multiple comparisons were found to be not significant, *P  >  *0.05.

Prior to evaluating GBS interactions with host and other microbial factors, we first characterized the impact of CAMP factor on GBS physiology under standard laboratory conditions. Using the GBS wild type (WT) serotype III, sequence type (ST) 17 clinical isolate COH1, and its isogenic *cfb* mutant generated previously (Δ*cfb*) (33), we assessed the impact of CAMP factor deficiency on GBS growth and morphology. We observed no differences between COH1 and Δ*cfb* growth in bacteriologic medium (Fig. 1A) nor did we observe any differences in Gram-staining or morphology under brightfield microscopy at mid-log phase (Fig. 1B).

Formation of biofilms is linked to virulence and colonization across bacterial species including GBS (45, 46). GBS biofilms can be disrupted by protease treatment (47) suggesting that surface and/or secreted proteins are involved in this process. Because CAMP factor is a secreted protein, and most highly expressed in late log phase (48), we evaluated whether CAMP factor contributes to GBS biofilm formation. We observed no differences in GBS biofilm formation between WT and Δ*cfb* strains as quantified by crystal violet staining (Fig. 1C). To test whether CAMP factor played a role in biofilm formation under stress conditions such as nutrient depletion or presence of secreted microbial factors, we assessed GBS biofilm formation between WT and Δ*cfb* strains grown in spent medium from stationary cultures of either self (WT COH1 and Δ*cfb*) or competitor methicillin-resistant S. aureus strain USA300 LAC as described in Materials and Methods. We observed no differences in GBS biofilm formation between WT and Δ*cfb* strains grown in spent medium from self or competitor conditions (Fig. 1D).

Difficulties in recombinant expression of GBS CAMP factor on high copy number plasmids in prior work has led to a hypothesis that there is a role for CAMP factor as a bacterial target *in vivo* (20). To determine whether CAMP factor contributed to microbe-microbe competition between GBS and S. aureus, we cocultured WT COH1 and Δ*cfb* with S. aureus USA300 LAC for 4 h and 24 h in Todd Hewitt Broth and determined viable CFU by serial dilution and plating. Although GBS outcompeted S. aureus under these conditions (competitive index  > 1), we observed no differences in competitive indices between COH1 and Δ*cfb* strains (Fig. 1E). Together, these results suggest that CAMP factor does not contribute to GBS growth, biofilm formation, or competition with S. aureus under laboratory experimental conditions.

### CAMP factor deficiency does not modify GBS adherence, invasion, or toxicity in immortalized human vaginal epithelial cells.

**FIG 2:**
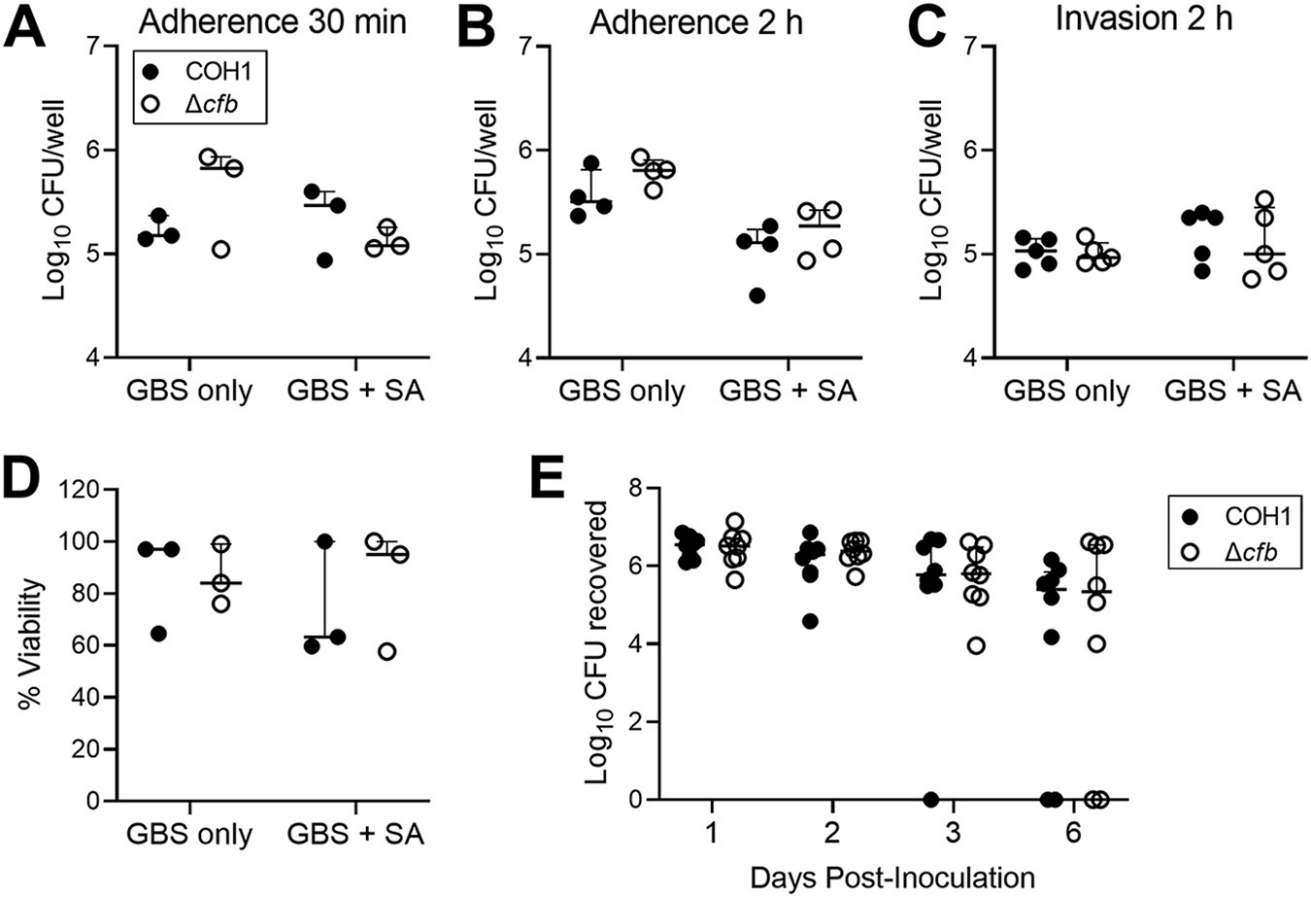
The role of GBS CAMP factor in vaginal epithelial colonization, adherence, and invasion. GBS (COH1 or Δ*cfb*) adherence to VK2 cells alone or in competition with S. aureus (SA) after 30 min (A) or 2 h (B) represented as recovered CFU per well. (C) GBS invasion of VK2 cells alone or in competition with SA after 2 h represented as recovered CFU per well. (D) VK2 cell viability GBS invasion alone or in competition with SA as determined by trypan blue exclusion. (E) WT C57BL/6J female mice were vaginally administered 2 × 10^7^ CFU of COH1 or Δ*cfb*. Mice were vaginally swabbed on days 1, 2, 3, and 6 postinoculation and the levels of GBS CFU recovered are shown. All *in vitro* experiments were performed in technical duplicates with three to five independent experimental replicates. *In vivo* GBS vaginal colonization (E) was performed once with eight mice per group. Individual points represent independent replicates (A to D) or biologic replicates (E) and lines showing median with interquartile range. All data were analyzed by two-way repeated measures ANOVA with Sidak’s multiple comparisons post-test. All multiple comparisons were found to be not significant, *P  >  *0.05.

Adherence to the host epithelium is a widely appreciated critical factor for mucosal colonization (49). Because prior work has identified a role for CAMP factor in S. pyogenes adhesion to a human pharyngeal cell line (50), we investigated whether GBS CAMP factor facilitates adherence to the vaginal epithelium. To test the role of GBS CAMP factor in adherence to human vaginal epithelial (VK2) cells, COH1 or Δ*cfb* were added to VK2 cell monolayers at a multiplicity of infection (MOI) of 10 and incubated for 30 min or 2 h prior to washing, lysing, and plating cells. No differences in VK2 adherence were observed between COH1 and Δ*cfb* at either 30 min (Fig. 2A) or 2 h (Fig. 2B). To test whether S. aureus would augment the contribution of CAMP factor to VK2 interactions, adherence assays were also conducted in the presence of S. aureus (SA) USA300 LAC added at the same time as GBS (GBS + SA). No differences in GBS adherence to VK2 cells were observed between COH1 and Δ*cfb* in the presence of S. aureus at 30 min or 2 h (Fig. 2A and B, respectively). However, at the 2 h time point, GBS adherence was reduced in the presence of S. aureus compared with GBS only conditions (*P  = * 0.0035, two-way ANOVA), but this effect was seen with both COH1 and Δ*cfb* strains (Fig. 2B). No differences in S. aureus adherence or invasion between were observed between the absence (SA only) or presence of COH1 and Δ*cfb* (GBS + SA) under any of these conditions (Fig. S1A to C) Additionally, we observed no differences between COH1 and Δ*cfb* adherence to human bladder epithelial (HTB-9) cells in the presence or absence of S. aureus (Fig. S1D).

Invasion of epithelial barriers is considered an important aspect of GBS dissemination (14, 51). Using invasion assays established previously (14, 51), VK2 cells were infected with GBS with or without S. aureus for 2 h, followed by 2 h of antibiotic treatment to kill extracellular bacteria. We observed no differences between COH1 and Δ*cfb* invasion of VK2 cells either in the presence, or absence of S. aureus (Fig. 2C). P. acnes CAMP factor has been shown to have cytotoxic properties against keratinocyte (HaCaT) and macrophage (RAW264.7) cell lines (52). Thus, we investigated whether GBS CAMP factor reduced cell viability of VK2 cells in the absence or presence of S. aureus. Similar to invasion conditions, VK2 cells were infected with GBS with or without S. aureus for 2 h followed by 2 h of antibiotic treatment, and viability was assessed by uptake of trypan blue. We observe no differences in cell viability between COH1 and Δ*cfb* in the presence or absence of S. aureus (Fig. 2D).

### GBS CAMP factor is dispensable for vaginal colonization in a murine model.

To determine the contribution of GBS CAMP factor production for colonization of the vaginal tract, we challenged 10-week-old C57BL/6J female mice (*n  *=  8 per group) with 10^7^ CFU of either COH1 or Δ*cfb* by intravaginal inoculation as described previously (53). Vaginal swabs were collected on days 1, 2, 3, and 6 postinoculation and GBS burdens quantified by plating on selective media. Although GBS burdens decreased over time (*P  = * 0.0083, two-way ANOVA), no differences in GBS burden occurred between COH1 and Δ*cfb* at any time point. Additionally, we did not observe any differences in GBS survival between COH1 and Δ*cfb* in mouse whole blood (Fig. S2).

## DISCUSSION

As an opportunistic pathogen, GBS is equipped with a variety of factors that facilitate mucosal colonization including surface adhesins and pili (54, 55), regulatory components (14, 56–60), and virulence factors promoting uterine dissemination such as the β-hemolysin/cytolysin toxin (15, 16) and hyaluronidase (61). Here, using similar *in vitro* and *in vivo* model systems, we were unable to establish a definitive contribution of CAMP factor at the vaginal mucosa. Prior studies indicated that CAMP factor may play a role in this environment. In RNA-sequencing analyses, GBS strain A909 CAMP factor (SAK_1983) expression is increased >7-fold in the mouse vaginal tract (59) and NEM316 strain CAMP factor (gbs2000) is >3-fold upregulated in human amniotic fluid (62) compared with laboratory medium. Additionally, GBS mutants deficient in the global transcriptional regulator CovRS, a critical factor for GBS vaginal colonization in mice (14), display markedly reduced *cfb* expression suggesting tight regulation of this virulence factor by GBS (63).

Our phenotypic analyses did not reveal any substantial deficits in the CAMP factor-deficient Δ*cfb* strain. CAMP factor is highly expressed in bacteriologic medium, peaking in late log phase, and is localized primarily to the cytoplasm and cell envelope (48) and the CAMP factor promoter has been used to stimulate high levels of eGFP expression in GBS (64). We observed no defects in GBS growth over 6 h or morphology in the Δ*cfb* strain at mid-log phase, although other time points were not evaluated in this study. Biofilm formation in GBS has been attributed to several GBS surface components including type 2a pili (46), biofilm regulatory protein A (57), and the GBS capsule production (47). We detected no significant differences in biofilm formation between WT and Δ*cfb* strains, supporting that CAMP factor is not involved in biofilm formation in GBS. Although S. aureus sphingomyelinase C (β-toxin) plays an important role in biofilm formation through its DNA ligase activity (65), we observed no contribution of S. aureus supernatant to GBS biofilm formation in the presence or absence of CAMP factor. Prior studies observed difficulties in successful heterologous CAMP factor expression in other bacterial species suggesting potential intracellular antimicrobial activity, however, no antimicrobial activity of purified CAMP factor was observed toward Escherichia coli or Bacillus subtilis (20). Likewise, we failed to detect any role for CAMP factor in GBS interspecies competition with S. aureus.

Bacterial contact with the host epithelium is a critical process for mucosal colonization, yet we observed no role of GBS CAMP factor in adherence or invasion of human vaginal epithelial cells for either GBS or S. aureus. As a limitation, our *in vitro* assays showed some variability across experimental replicates; thus, our findings may be underpowered to observe subtle effects. Similar to our findings, no role for GBS CAMP factor was established for adherence or invasion of human brain microvascular endothelial cells (33). Additionally, S. pyogenes CAMP factor enhances adherence and invasion of human pharyngeal carcinoma cells in a serum-independent manner, but not human lung or keratinocyte cells (50). Moreover, CAMP factor deficiency in P. acnes did not alter transcriptional responses of keratinocyte cells to P. acnes infection (66). Together, these results suggest that CAMP factor is dispensable for GBS interactions with the vaginal epithelium and support the limited role for CAMP factors in mucosal colonization across bacterial species.

CAMP factor-dependent macrophage cytotoxicity has been observed during P. acnes coinfection with S. aureus (67), but we did not observe any evidence for cytotoxicity of GBS CAMP factor in vaginal epithelial cells in the presence or absence of S. aureus. Our findings may in part be explained by differences in cell membrane lipid composition of epithelial cells versus blood or immune cells. Red blood cell membranes with at least 45% sphingomyelin (sheep, cow, goat) are susceptible to CAMP factor lysis, while species with lower levels of red blood cell sphingomyelin, such as human, mouse, and rabbit, are not susceptible (19). Similar to our findings, no cytotoxicity of S. pyogenes CAMP factor was observed in human pharyngeal carcinoma cells (50) or murine macrophages (32). To our knowledge, sphingolipid concentrations in vaginal epithelial cells is undefined. In a clinical study, cervicovaginal sphingomyelin levels were positively correlated with genital inflammation and vaginal pH, although specific microbial signatures associated with increased sphingomyelin were not observed (68). Contribution of microbial sphingomyelinases, such as from S. aureus (65) or Trichomonas vaginalis (69), to interspecies cross-feeding and stimulation of host inflammation in the vaginal environment should be addressed in future studies.

There are several limitations to our study. While GBS CAMP factor (SAN_2173) is more than 98% identical at the nucleotide level across sequenced GBS isolates using NCBI BLASTN 2.12.0 (70), a second GBS CAMP factor, CAMP factor II (SAL_2074, SAN_2140), was recently identified on an integrative and conjugative element within certain GBS strains including the COH1 strain included in our experiments with ∼73% homology to CAMP factor (71). Although a CAMP test of COH1 and the *cfb* mutant used in this study showed no CAMP activity in the *cfb* mutant (33), compensation of this second CAMP factor II in our assays cannot be ruled out. Additionally, the CAMP test is temperature dependent and occurs most optimally at 15°C to 30°C (19) and thus is not reflective of animal or human environments, except for perhaps the skin. Host acid sphingomyelinase enhances cytotoxic and inflammatory properties of P. acnes CAMP factor *in vitro* and in skin infection models (52). However, we did not quantify host sphingomyelinase in our assays. Moreover, we did not add exogenous sphingomyelinase to our assay conditions, but instead coincubated cells with S. aureus, or used conventional mice colonized with microbiota to assess the role of GBS CAMP factor in models most reflective of GBS vaginal colonization. Additionally, we did not collect samples beyond 6 days postinoculation (Fig. 2E) and thus cannot rule out differences at later time points.

An additional limitation of this study is that we did not assess the role of GBS CAMP factor in polymicrobial infections with S. aureus in an invasive context such as sepsis, which is beyond the scope of this work. Although murine models have been useful in identifying GBS interactions with other species such as Gardnerella vaginalis (72) and Proteus bivia (73), it is possible that GBS CAMP factor plays a role in human vaginal colonization via a mechanism not present in our murine model. C57BL/6J mice, such as those used in our study, typically have Staphylococcus succinus as a dominant member of the vaginal microbiota (74). A recent whole genome sequence analysis of an S. succinus isolate from fermented soybeans did not reveal any predicted sphingomyelinases present (75, 76), and we speculate this may result in lower sphingomyelinase activity and subsequent lack of co-hemolytic CAMP factor activity in the murine vagina. Global transcriptional profiling of S. aureus collected from mouse vaginal swabs identified a 2-fold increased expression in sphingomyelinase C (locus SAUSA300_1973) at day 3 postinoculation compared with tryptic soy broth medium but no differences at earlier time points (77). It is currently unknown whether sphingomyelinase C is important for vaginal colonization in humans or murine models. Thus, our results do not exclude the possibility that GBS CAMP factor is required for colonization or infection in humans.

In summary, we conclude that, although the expression of GBS CAMP factor remains a widely used assay in clinical microbiology, our results using *in vitro* human cell lines and murine models do not support a critical role for CAMP factor in promoting GBS vaginal colonization.

## MATERIALS AND METHODS

### Bacterial strains and growth conditions.

Streptococcus agalactiae strains used in this study were GBS COH1 (ATCC BAA-1176) and isogenic COH1 Δ*cfb* generated by allelic exchange with chloramphenicol acetyltransferase (*cat*) antibiotic resistance gene as described previously (33). Community-acquired methicillin-resistant Staphylococcus aureus USA300 LAC (78) was also used. Bacteria were grown in Todd Hewitt Broth (THB, Hardy Diagnostics) or THB agar plates at 37°C without shaking. Overnight cultures were diluted in fresh THB and incubated at 37°C until mid-logarithmic phase (defined as OD_600_ = 0.4). For growth curves, stationary cultures were diluted 1:100 in fresh THB and incubated at 37°C for 6 h with optical density (OD_600_) measured every 30 min. Log phase cultures were subjected to a standard Gram-staining (BD BBL), protocol and bright-field images collected under oil immersion at 630× magnification on an Echo Revolve microscope.

### Human cell lines and growth conditions.

VK2 (catalog no. CRL-2616; ATCC) were grown in keratinocyte serum-free medium (KSFM) supplemented with human recombinant epidermal growth factor and bovine pituitary extract (Life Technologies), and bladder epithelial cells (HTB-9; catalog no. 5637; ATCC) were grown in Roswell Park Memorial Institute 1640 (RPMI 1640, Gibco) medium with 10% fetal bovine serum. Cell lines were cultured at 37°C with 5% CO2 and passages 5 to 25 were used for experiments.

### Biofilm assays.

GBS biofilms were assessed by crystal violet staining as adapted from prior methods (57). Overnight cultures of GBS and S. aureus grown in THB were pelleted and supernatant was syringe filtered. Bacterial pellets were resuspended in fresh THB, brought to OD_600_ = 0.2, and mixed 1:1 with either fresh THB, self-supernatant, or competitor supernatant in tissue culture-treated 96-well plates at a final volume of 200 μl. Biofilms were allowed to form for 24 h at 37°C without shaking. After washing 3× with PBS, biofilms were dried at 55°C for 30 min and stained with 0.2% crystal violet for 15 min. Biofilms were washed 3× with PBS and destained with an 80:20 mixture of ethanol:acetone. Supernatants were transferred to a new 96-well microtiter plate and absorbance at 595 nm was measured on a BioTek Cytation 5 multi-mode plate reader.

### Co-culture assays.

Stationary GBS (COH1 or Δ*cfb*) and S. aureus (SA) were grown in THB to mid-logarithmic phase and diluted 1:100 in fresh THB either as single cultures or cocultures of GBS + SA. Cultures were serial diluted and plated on THB agar at 0 h, 4 h, and 24 h. GBS strains and S. aureus were distinguished by colony morphology and pigmentation (S. aureus, larger yellow colonies; GBS, smaller white colonies). Competitive index was calculated as the ratio of recovered GBS strain CFU/mL to SA CFU/mL.

### Adherence assays.

Vaginal and bladder epithelial adherence assays were performed as adapted from prior methods (57, 79). Briefly, VK2 or HTB9 cells were grown to confluent monolayers in 24-well tissue culture plates. Subsequently, cells were infected with COH1 or Δ*cfb* at a multiplicity of infection (MOI; calculated as the GBS to cell ratio) of 10 alone, or in combination with MOI of 10 of S. aureus. Bacterial-cell contact was facilitated by centrifugation for 1 min at 200 × *g*. Cells were then incubated at 37°C in 5% CO_2_ for 30 min or 2 h as indicated in figure legends, supernatant was removed, and the cells were washed 6× with sterile PBS. Cells were incubated with 100 μL 0.25% trypsin/2.21 mM EDTA for 5 min and then 400 μL of 0.025% Triton-X in PBS was added to each well and cells were pipetted vigorously 30× to ensure detachment and lysis. Bacterial recovery was determined by plating samples on THB agar. GBS and S. aureus colonies were distinguished as above. Data was expressed as a percentage of adherent CFU compared to original inoculum.

### Invasion assays.

Confluent VK2 monolayers were infected with COH1 or Δ*cfb* at an MOI of 10 alone, or in combination with MOI of 10 of S. aureus. Bacteria were brought into contact with the VK2 cells by centrifugation for 1 min at 200 × *g*. Cells were incubated at 37°C in 5% CO_2_ for 2 h, supernatant was removed, and cells were washed 3× with sterile PBS. Gentamycin (100 μg/mL) and Penicillin (20 μg/mL) were added in 500 μL of fresh KSFM, and cells were incubated at 37°C in 5% CO_2_ for 2 additional hours. Cells were washed 3× with PBS, followed by detachment and lysis with 0.25% trypsin/2.21 mM EDTA and 0.025% Triton-X in PBS as described in adherence assays. Bacterial recovery was determined by plating on THB agar and data was expressed as a percentage of invasive CFU compared with original inoculum. VK2 viability was assessed via trypan blue exclusion. At the 4 h invasion assay endpoint, cells were treated with 50 μL of 0.25% trypsin/2.21 mM EDTA for 5 min at 37°C. RPMI 1640 with 10% FBS (150 μL/well) was added to inactivate the trypsin. To distinguish live cells from dead cells, cells were mixed 1:1 with 0.4% trypan blue, and live cells (unstained) and dead cells (blue) were enumerated using a hemocytometer and visualization on a Leica DMi1 microscope. Percent of live cells per sample was calculated as (number of live cells/total cells counted) × 100.

### Animals.

The BCM Institutional Animal Care and Use Committee approved all animal protocols and procedures. Wild type (WT) C57BL/6J mice aged 8 to 12 weeks were purchased from Jackson Laboratories (strain code 000664). Female mice were used for vaginal colonization experiments and both male and female mice were used for whole blood survival experiments. Groups were assigned randomly and housed at four animals per cage in separate cages. Mice could eat and drink *ad libitum*.

### *In vivo* GBS vaginal colonization.

Vaginal colonization studies were conducted as previously described (53). Briefly, mice were synchronized with 0.5 mg of β-estradiol administered i.p. 24 h prior to inoculation. Mice were inoculated with 10 μL (1 × 10^7^ CFU) of GBS COH1 or Δ*cfb* into the vaginal tract. The vaginal lumen was swabbed daily for 3 days postinoculation and again on day 6. Recovered GBS was quantified by plating on CHROMagar StrepB (DRG International).

### Whole blood survival.

Stationary phase COH1 and Δ*cfb* bacteria were diluted 1:10 in PBS and 10 μL of diluted bacteria was added to 90 μL of heparin-treated murine blood in nonbinding 96-well plates (Costar 3641). After a 30-min incubation at 37°C with gentle rotation, blood samples were serially diluted and plated to enumerate GBS survival. GBS survival was determined by plating samples on THB agar and presented as recovered CFU/mL of whole blood.

### Statistical analyses.

All *in vitro* experiments were performed in at least technical duplicate and repeated in at least three independent replicate experiments. *Ex vivo* whole blood survival assays were performed in technical duplicate with five biologic replicates, and the *in vivo* vaginal colonization model was performed as one independent experiment with eight biological replicates per group. Mean values derived from technical replicates were utilized for statistical analyses. When sample size was too small to determine normality, data were assumed nonparametric and thus assessed with nonparametric statistical analyses. Statistical analyses were conducted using GraphPad Prism, version 9.1.2 (GraphPad Software Inc., La Jolla, CA) and *P values* of  <0.05 were considered statistically significant. Growth curves, coculture competition, adherence and invasion experiments, cell viability, and *in vivo* vaginal colonization were analyzed by two-way repeated measures ANOVA with Sidak’s multiple-comparison test. Biofilm formation in the presence of supernatants were analyzed by two-way repeated measures ANOVA with Tukey’s multiple-comparison test. Biofilm formation in the absence of bacterial supernatants and whole blood killing were analyzed by Wilcoxon matched-pairs signed rank test.
