# Supplementary material for: Group B Streptococcus CAMP Factor Does Not Contribute to Interactions with the Vaginal Epithelium and Is Dispensable for Vaginal Colonization in Mice
Source: Microbiol Spectr. 2021 Dec 15;9(3):e01058-21. doi: 10.1128/Spectrum.01058-21 (PMC8672899; doi:10.1128/Spectrum.01058-21)
Supplement: SUPPLEMENTAL FILE 1 — Supplemental material. Download SPECTRUM01058-21_Supp_1_seq6.pdf, PDF file, 0.4 MB [file spectrum01058-21_supp_1_seq6.pdf]

Group B Streptococcus CAMP factor does not contribute to interactions with the vaginal epithelium and is dispensable for vaginal colonization in mice

Mallory B. Ballard, Vicki Mercado-Evans, Madelynn G. Marunde, Hephzibah Nwanosike, Jacob Zulk, Kathryn A. Patras

SUPPLEMENTAL DATA AND LEGENDS

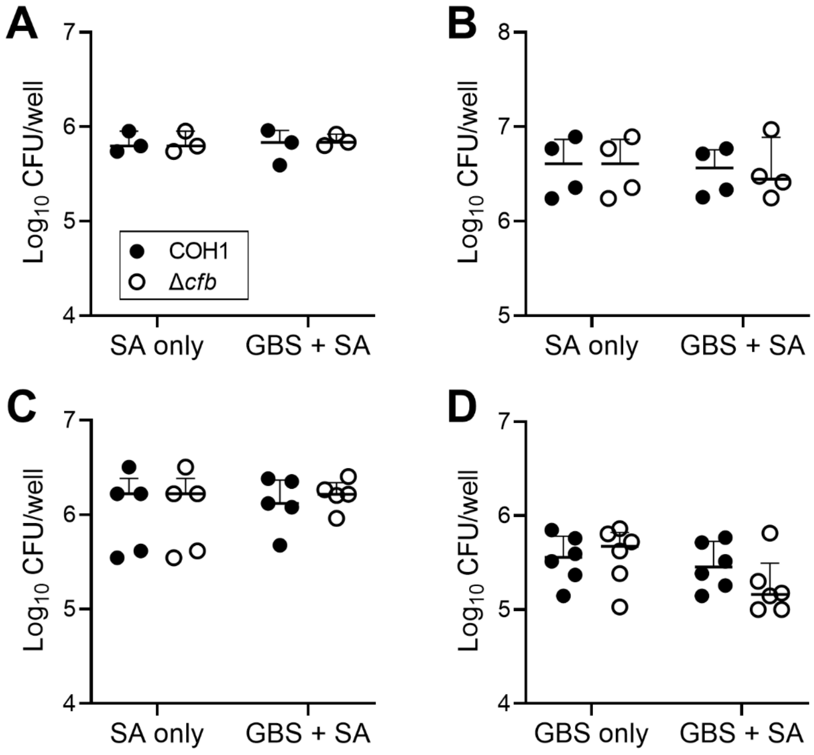

Supplemental Figure 1: GBS CAMP factor deficiency does not alter *S. aureus* adherence of invasion of VK2 cells nor GBS adhesion to human bladder epithelial cells. *S. aureus* USA300 LAC (SA) adherence to VK2 cells alone or in competition with GBS COH1 or Δ*cfb* after 30 minutes (**A**) or 2 h (**B**) represented as recovered CFUs per well. **C)** *S. aureus* invasion of VK2 cells alone or in competition with GBS COH1 or Δ*cfb* after 2 h represented as recovered CFUs per well. **D**) GBS (WT COH1 or Δ*cfb*) adherence to HTB-9 cells alone or in competition with *S. aureus* (SA) after 2 h incubation represented as recovered CFUs per well. SA only values (A-C) are identical values between COH1 and and Δ*cfb* groups (no GBS present) and are shown merely for comparison to (GBS + SA) conditions. Experiments were performed in technical duplicates with 6 independent experimental replicates. Individual points represent independent replicates and lines showing median with interquartile range. Data were analyzed by two-way repeated measures ANOVA with Sidak's multiple comparisons post-test comparisons were found to be not significant, $P > 0.05$.

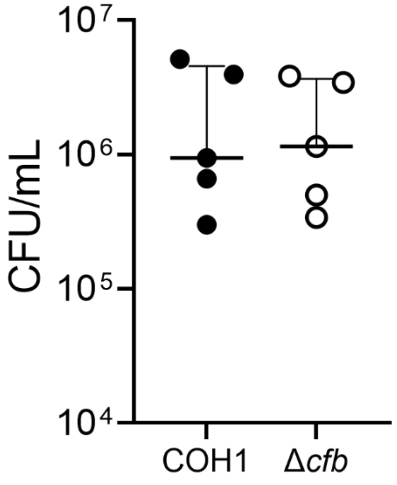

Supplemental Figure 2: GBS CAMP factor deficiency does not alter GBS survival in murine whole blood. GBS COH1 or Δ*cfb* survival after 30 min incubation in murine whole blood expressed as recovered CFU/mL of blood. Experiments were performed in technical duplicates with 5 biological replicates. Individual points represent independent replicates and lines showing median with interquartile range. Data were analyzed by Wilcoxon matched-pairs signed rank test, and comparisons were found to be not significant, $P > 0.05$.
